# Fat Content Modulates Rapid Detection of Food: A Visual Search Study Using Fast Food and Japanese Diet

**DOI:** 10.3389/fpsyg.2017.01033

**Published:** 2017-06-22

**Authors:** Reiko Sawada, Wataru Sato, Motomi Toichi, Tohru Fushiki

**Affiliations:** ^1^Department of Neurodevelopmental Psychiatry, Habilitation and Rehabilitation, Graduate School of Medicine, Kyoto UniversityKyoto, Japan; ^2^Organization for Promotion of Neurodevelopmental Disorder ResearchKyoto, Japan; ^3^Faculty of Human Health Science, Graduate School of Medicine, Kyoto UniversityKyoto, Japan; ^4^Faculty of Agriculture, Ryukoku UniversityOtsu, Japan

**Keywords:** rapid detection of food, fat content, fast food, Japanese diet, visual search

## Abstract

Rapid detection of food is crucial for the survival of organisms. However, previous visual search studies have reported discrepant results regarding the detection speeds for food vs. non-food items; some experiments showed faster detection of food than non-food, whereas others reported null findings concerning any speed advantage for the detection of food vs. non-food. Moreover, although some previous studies showed that fat content can affect visual attention for food, the effect of fat content on the detection of food remains unclear. To investigate these issues, we measured reaction times (RTs) during a visual search task in which participants with normal weight detected high-fat food (i.e., fast food), low-fat food (i.e., Japanese diet), and non-food (i.e., kitchen utensils) targets within crowds of non-food distractors (i.e., cars). Results showed that RTs for food targets were shorter than those for non-food targets. Moreover, the RTs for high-fat food were shorter than those for low-fat food. These results suggest that food is more rapidly detected than non-food within the environment and that a higher fat content in food facilitates rapid detection.

## Introduction

Efficient visual attention to food is crucial for organisms. Due to limitations in human information processing, detecting food entails prioritizing significant signals from the environment (Tooby and Cosmides, 1990). Humans benefit if they can find food rapidly in their environment, because survival depends on the ability to maintain energy and health, which are, in turn, dependent on diet. Thus, it has been proposed that the visual system evolved and was shaped to efficiently orient attention toward food (Spence et al., 2016).

However, empirical evidence supporting the idea of rapid detection of food remains inconsistent. Previous experimental psychological studies have examined this issue using the visual search paradigm (Nummenmaa et al., 2011; de Oca and Black, 2013). The visual search paradigm has been successfully applied to demonstrate the ability of the human visual system to detect important signals in the environment. Researchers arranged photographs of food and/or non-food and asked participants to respond regarding the existence of a different stimulus (i.e., target) from other stimuli (i.e., distractors). Nummenmaa et al. (2011) showed that reaction times (RTs) for detecting a food target among non-food distractors are shorter than those for detecting a non-food target among food distractors using appetizing and plain food items and cars as target stimuli; however, in another experiment, they showed no clear difference in RTs for detecting food vs. non-food targets when non-food stimuli were visually similar to food stimuli. de Oca and Black (2013) also showed that a food target was detected faster than a non-food target (i.e., flowers); however, they failed to show any advantage in detection speed for food when they compared RTs for a food target with those for the other type of non-food target (i.e., chairs). Thus, previous visual search studies have reported inconsistent results regarding more rapid detection of food vs. non-food targets. Additionally, the difference in speed of detection of food vs. non-food targets is difficult to quantify because the search symmetry paradigm (Treisman and Souther, 1985) was used in some previous experiments (Nummenmaa et al., 2011; de Oca and Black, 2013). In the search symmetry paradigm, researchers compare the detection RTs of a food target among non-food distractors vs. those of a non-food target among food distractors, even in cases where they showed that the food targets were detected more rapidly than the neutral non-food targets. Because detection RTs in this method reflect both target and distractor effects, it was pointed out that visual attention captured by a target cannot be distinguished from serial scanning of a crowds of distractors (Eastwood et al., 2001; Horstmann and Bauland, 2006). Evidence was also provided by studies using different experimental paradigms, in which visual attention was greater toward food stimuli (Nijs et al., 2010; Harrar et al., 2011; Kumar et al., 2016). For example, Kumar et al. (2016) reported shorter RTs for neutral targets (e.g., a circle) presented in the same direction of food stimuli vs. those for targets presented in the same direction of non-food stimuli (e.g., cars). Thus, although some experimenters reported that food is more rapidly detected than non-food (Nummenmaa et al., 2011; de Oca and Black, 2013), which is in accordance with the results obtained for different experimental paradigms (e.g., Kumar et al., 2016), the evidence has not yet resulted in a definitive conclusion.

Furthermore, the effect of dietary fat content on visual attention toward food remains unclear. It is well known that dietary fat allows maintenance of bodily functions but also efficient energy intake (i.e., high energy density, Drewnowski, 1998). Humans can accurately evaluate fat content based only on visual information about food without actually ingesting it (Toepel et al., 2009). To ingest energy efficiently and thus achieve a survival benefit, visual attention may be modulated according to the perceived fat content of food. Consistent with this notion, experimental research has revealed an effect of fat content on visual processing. For example, a behavioral study showed that non-food targets following images of high-fat food were found faster than those following images of low-fat food (Harrar et al., 2011). Another study measuring eye movements reported that high-fat food was more frequently fixated on first and gazed at for longer than were non-food items (Garcia-Burgos et al., 2017). Moreover, an electrophysiological study showed differences in the brain activity associated with visual processing of biologically salient stimuli between responses to images of high-fat and those to low-fat food (Toepel et al., 2009). These results suggest that high-fat food captures visual attention more readily than low-fat food. However, to the best of our knowledge, no visual search study has investigated the effects of fat content of food on the speed of detecting food images. Thus, although some studies have shown that fat content can affect visual attention, it remains unknown whether the fat content of food modulates detection.

In the present study, we investigated the effect of food and its fat content on rapid detection using a visual search paradigm. Photographs of fast food, the Japanese diet, and non-food items were used as target stimuli. We selected fast food (e.g., hamburger) as the high-fat food stimuli, as it is known to contain high levels of fat, although the origin of the name is to fill the stomach as quickly as possible, thereby efficiently saving time. We also selected items representative of the Japanese diet (e.g., sushi) as the low-fat food stimuli, because such items are known to contain low levels of fat. Frequent consumption of fast food leads to weight gain (Pereira et al., 2005). Moreover, the dietary change from the Japanese to an American diet, which entails increased levels of fat intake, increases the risk of obesity and diseases, such as coronary heart disease and cancer (Shimizu et al., 1991; Ueshima et al., 2003). Based on previous experiments reporting that food is more rapidly detected than non-food (Nummenmaa et al., 2011; de Oca and Black, 2013), we predicted that both fast food and the Japanese diet items would be detected faster than non-food targets. Moreover, given the enhanced visual attention afforded to food with high levels of fat (Toepel et al., 2009; Harrar et al., 2011), we also predicted that fast food would be detected faster than the Japanese diet items. In addition, we assessed the possible effects of body mass index (BMI), self-reported hunger levels, subjective liking, and evaluations of the monetary value of food on the speed of detection of food targets, because these factors could affect the visual processing of food (Nijs et al., 2010; Tapper et al., 2010; Nummenmaa et al., 2011; de Oca and Black, 2013; Bielser et al., 2016; for a review, see Werthmann et al., 2015).

## Materials and Methods

### Participants

Thirty-two Japanese participants (16 females, mean ±*SD* age = 23.28 ± 4.92 years) participated in this study. The sample size of this study was determined by *a priori* power analysis, done using G^∗^Power software (ver. 3.1.9.2; Faul et al., 2007, 2009). We analyzed RTs using a repeated-measures analysis of variance (ANOVA) with one within-participants factor (three levels), with an *α* of 0.05, power (1-β) of 0.80 (Cohen, 1988), and *𝜀* of 0.5 (Faul et al., 2007). Because the effect size was unclear, we predicted medium-sized effects (*f* = 0.25) (Faul et al., 2007). The result indicated that more than 29 samples were necessary.

All participants had normal or corrected-to-normal visual acuity. The BMI of all participants fell within the normal range (18.50–24.99 kg/m^2^; mean ±*SD* = 21.06 ± 1.74). Prior to the experiment, the participants confirmed that they did not have any food intake restrictions and they were instructed to refrain from eating for more than 3 h before coming to the laboratory. The study was carried out in accordance with the guidelines of the Ethics Committee of the Graduate School of Medicine, Kyoto University with written consent obtained from all participants in accordance with the Declaration of Helsinki. The protocol was approved by the Ethics Committee of the Graduate School of Medicine, Kyoto University.

### Stimuli

**FIGURE 1:**
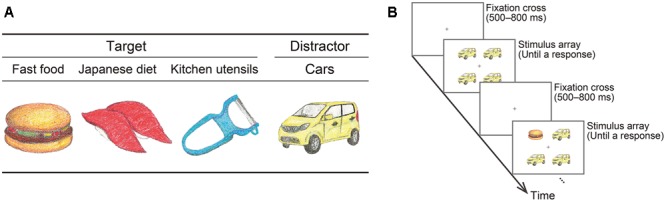
Schematic illustration of stimuli **(A)** and visual search task **(B)**. Actual stimuli were full-color photographs. The examples of fast food, Japanese diet items, and kitchen utensils consist of a hamburger, sushi, and a peeler, respectively.

Schematic illustrations of example stimuli are shown in **Figure 1** (see **Supplementary Figure S1** for illustrations of all stimuli); note that actual stimuli were full-color photographs. The food target stimuli were five fast food items (i.e., a hamburger, slice of pizza, fried chicken, fried potatoes, and donut) and five items from the Japanese diet [i.e., sushi (sushi with a topping of tuna), udon (Japanese wheat noodles), yakitori (Japanese grilled chicken), niku-jaga (simmered meat, potatoes, onions, and so on), and manju (a bun with a bean-jam filling)]. Photographs of five kitchen utensils (i.e., a peeler, can opener, kettle, frying pan, and scourer) were also used as non-food target stimuli to compare RTs for detecting food vs. non-food targets. The distractor stimuli were full-color pictures of five types of car, as per a previous visual search study (Nummenmaa et al., 2011). The original pictures of all items were taken from websites of several companies, such as restaurants, stores, and automakers. Using Photoshop software (ver. CS6; Adobe), we extracted each item from its background and placed it on a white square. According to ANOVAs with type (fast food, Japanese diet, kitchen utensils and cars), mean luminance [*F*(3,16) = 0.94, *p* = 0.45, $\eta_{p}^{2}$ = 0.15], contrast (Michelson, 1927) [*F*(3,16) = 2.91, *p* = 0.07, $\eta_{p}^{2}$ = 0.35], and RGB scores [*F*(3,16) = 0.62, *p* = 0.61, $\eta_{p}^{2}$ = 0.11], which were obtained using Photoshop software (ver. CS6; Adobe), did not differ among the stimulus categories.

Information concerning calories and the amount of protein, fat, carbohydrates, and salt in food items per image was obtained from the menus and websites that provided the original pictures of stimuli, and is shown in Supplementary Table S1. According to *t*-tests using Welch’s method (Welch, 1938, 1947), no differences in protein, carbohydrate, salt, or caloric contents were detected between fast food and the Japanese diet items [*t*(8) = 0.31, 0.36, 0.32, and 0.87 *p* = 0.77, 0.73, 0.76, and 0.41, for protein, carbohydrate, salt, and caloric contents, respectively], but the amount of fat in fast food was higher than that in the Japanese diet items [*t*(8) = 2.77, *p* = 0.03].

To investigate emotional and familiarity aspects, we conducted an additional rating experiment with 16 participants (9 females), none of whom took part in the visual search experiment. We asked them to provide subjective emotional ratings of arousal and valence (i.e., the intensity and quality of the emotion that participants felt when perceiving the stimulus), and familiarity (i.e., the frequency with which they see the food item in daily life) using a 9-point scale ranging from 1 (“low arousal,” “negative,” and “unfamiliar”) to 9 (“high arousal,” “positive,” and “familiar”). The data are shown in Supplementary Table S2.

One-way repeated-measures ANOVAs with type (fast food, Japanese diet, kitchen utensils, and cars) as the within-participant factor showed significant main effects of type for arousal [*F*(3,45) = 24.33, *p* < 0.001, $\eta_{p}^{2}$ = 0.62] and valence [*F*(3,45) = 21.08, *p* < 0.001, $\eta_{p}^{2}$ = 0.58]. Multiple comparisons with Bonferroni’s correction showed that both fast food and Japanese diet items enhanced emotional arousal (*t*s > 4.83, *p*s < 0.001) and positive feelings (*t*s > 4.18, *p*s < 0.005) compared with kitchen utensils and cars. No differences in emotional ratings were detected between fast food and the Japanese diet items (*t*s < 1.25, *p*s > 0.99) or between kitchen utensils and cars (*t*s < 1.62, *p*s > 0.76). Regarding familiarity, a one-way repeated-measures ANOVA with type (fast food, Japanese diet, kitchen utensils, and cars) showed a significant main effect of type [*F*(3,45) = 9.93, *p* < 0.001, $\eta_{p}^{2}$ = 0.40]. Multiple comparisons with Bonferroni’s correction showed no difference in familiarity among fast food, the Japanese diet, and kitchen utensils (i.e., target stimulus categories) [*t*s < 1.95, *p* > 0.42], although these three target stimuli were more familiar than cars (*t*s > 3.21, *p* < 0.05). In summary, food stimuli enhanced positive emotions compared with non-food stimuli, whereas no difference in familiarity was found between fast food items, Japanese diet items, and kitchen utensils.

Subjective ratings of eating frequency for food stimuli (i.e., the frequency with which the participants consumed the food item in daily life) were also obtained using a 7-point scale ranging from 1 (“not at all”) to 7 (“very frequently”). The paired *t*-test showed that eating frequency was lower for fast food items than for Japanese diet items [*t*(15) = 2.63, *p* = 0.02].

Moreover, subjective ratings of healthiness (i.e., healthiness of each food as perceived by the participants) for fast food and the Japanese diet items were obtained using a 9-point scale ranging from 1 (“unhealthy”) to 9 (“healthy”). Consistent with general beliefs (cf. Japan External Trade Organization, 2013), a paired *t*-test showed that the Japanese diet items were rated as healthier than fast food [*t*(15) = 10.00, *p* < 0.001].

The stimuli were displayed in 2 × 2 arrays against a white background. An illustration of the stimulus display is shown in **Figure 1B**. Each stimulus subtended a visual angle of 3.07° × 3.07°. A black cross subtending a visual angle of 0.51° × 0.51° was presented as a fixation point at the center of the stimulus array. The array subtended a visual angle of 7.15° × 7.15°. To enable a simple and direct measurement of the rapidity with which food vs. non-food targets were detected, the distractors were constant (i.e., cars) across all target conditions (Eastwood et al., 2001; Horstmann and Bauland, 2006). Distractor stimuli were all the same (i.e., one type selected from five types of cars) in the display under the target present and target absent conditions.

### Apparatus

The presentation of stimuli was controlled by Presentation software (ver. 14.9; Neurobehavioral Systems) implemented on a Windows computer (HP Z200 SFF; Hewlett-Packard). The stimuli were presented on a 19-inch CRT monitor (HM903D-A; Iiyama) with a refresh rate of 150 Hz and a resolution of 1,024 pixels × 768 pixels. The response was obtained using a response box (RB-530; Cedrus), which measures RT with 2-3 ms resolution.

### Visual Search Task

The experiment was conducted in a soundproof room (Science Cabin; Takahashi Kensetsu) between 10:30 am and 7:00 pm. Participants sat in chairs with their chins fixed in a steady position 60 cm from the monitor. They were asked to keep their gaze on the fixation cross at the center of the display. Before the experiment began, participants completed 24 practice trials, which involved the same procedure as that used in the experiment, to gain familiarity with the apparatus.

The experiment consisted of a total of 240 trials, presented in four blocks of 60 trials, with an equal number of target-present and target-absent trials (i.e., 120 trials of each). In the target-present trials, the position of the target stimulus was randomly chosen, but they were presented in each position of the 2 × 2 array an equal number of times. All four stimuli in the target-absent trials were cars. Trial order was randomized across all conditions within a block. In each trial, the fixation cross was presented, and then the stimulus array consisting of four items was then presented until participants responded. Participants were asked to respond as quickly and accurately as possible by pushing the appropriate buttons using their left or right index finger to indicate whether all four items were the same, or one item was discrepant. The positions of the response buttons were counterbalanced across participants. The inter-stimulus interval varied from 500 to 800 ms.

Prior to beginning the visual search experiment, participants were asked to rate their level of hunger on a 5-point scale (1 = “very hungry,” 5 = “very full”). The majority of the participants felt hungry (very hungry: 25.00%, hungry: 59.38%; mean ±*SD* = 2.00 ± 0.76).

### Questionnaire

Following the visual search task, the participants completed questionnaires to assess possible confounding factors including BMI, self-reported hunger level, and subjective ratings of liking, because these factors could affect the visual processing of food. A previous visual search study reported that a search advantage for food, which was calculated by subtracting RTs for food targets among non-food distractors from those for non-food targets among food distractors, was negatively related to BMI (Nummenmaa et al., 2011). Several studies using a visual probe task showed that hunger enhanced visual attention to food stimuli (Nijs et al., 2010; Tapper et al., 2010). Bielser et al. (2016) also reported that the RTs of judgments concerning liking food were faster when the stimuli were an individual’s preferred food. Moreover, we assessed the evaluations of the monetary value of food items. A visual search study showed that monetary targets were detected more rapidly than neutral targets (e.g., a couch) (de Oca and Black, 2013), suggesting that money enhances visual attention by eliciting motivationally relevant pleasant emotions. In summary, it was revealed that individual differences in these possible confounding factors affected the degree of visual attention to food, although several studies reported inconsistent results regarding the effects of these factors on detecting food items, such as the null effect of hunger level on detecting food (Nummenmaa et al., 2011; de Oca and Black, 2013; for a review, see Werthmann et al., 2015).

In this study, the participants were presented with a list of the food items used in the visual search task and asked to rate in terms of the degree of liking on 5-point scales (1 = “dislike very much,” 5 = “like very much”). Participants were also asked whether they were allergic to any of the food, and to rate each food item with respect to their monetary value (i.e., indicate what they believed to be a reasonable price to pay for each food).

### Data Analyses

The mean RT and percentage of correct responses in target trials were calculated under each condition, excluding measurements ± 3 *SD* from the mean of target-present trials. As the primary performance measure in the visual search, we analyzed correct response RTs as in previous studies (Öhman et al., 2001; Sato and Yoshikawa, 2010; Sawada et al., 2016). The correct RTs and percentages were then subjected to one-way repeated-measures ANOVAs with type (fast food, Japanese diet, and kitchen utensils) as the within-participant factor. Multiple comparisons were conducted with Bonferroni’s method for better understanding of the significance of the main effects.

The mean subjective ratings of liking and price were calculated for fast food and the Japanese diet items, and these data were then subjected to paired *t*-tests. Moreover, to test the relationship between food detection RTs and other variables (BMI, hunger level, and subjective ratings), correlation analyses were conducted on data collected under the fast food and Japanese diet conditions.

Preliminarily, the gender difference in correct RTs and percentages was assessed by two-way ANOVAs with type (fast food, Japanese diet, and kitchen utensils) and gender (female and male). There was no significant main effect [*F*(1,30) = 0.49 and 1.19, *p* = 0.49 and 0.28, $\eta_{p}^{2}$ = 0.02 and 0.04, for RTs and accuracy, respectively] or interaction [*F*(1,30) = 1.31 and 0.17, *p* = 0.28 and 0.84, $\eta_{p}^{2}$ = 0.04 and 0.01, for RTs and accuracy, respectively] related to gender. Subjective ratings were also assessed by ANOVAs with type (fast food and Japanese diet) and gender (female and male) as the factors. No significant main effect [*F*(1,30) = 0.06 and 0.03, *p* = 0.81 and 0.87, $\eta_{p}^{2}$ = 0.002 and 0.01, for liking and monetary value, respectively] or interaction [*F*(1,30) = 0.78 and 1.50, *p* = 0.38 and 0.23, $\eta_{p}^{2}$ = 0.03 and 0.05, for liking and monetary value, respectively] related to gender was observed. Thus, gender was omitted in subsequent analyses.

## Results

### Reaction Time (RT)

**FIGURE 2:**
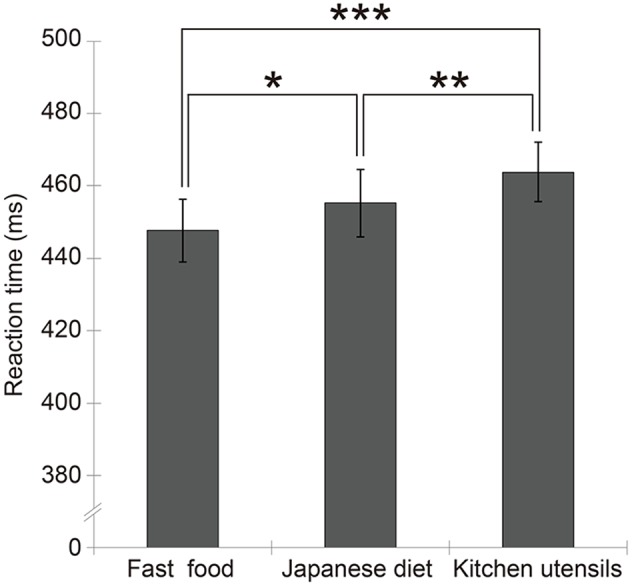
Mean (with ±*SE*) reaction time for each target condition. Significant results are shown by asterisks (^∗^*p* < 0.05, ^∗∗^*p* = 0.01, ^∗∗∗^*p* < 0.001).

Mean (±*SE*) RT under each target condition (fast food: 447.7 ± 8.6 ms; Japanese diet: 455.3 ± 9.3 ms; kitchen utensils: 463.8 ± 8.2 ms) is shown in **Figure 2**. The data of each participant is also shown in Supplementary Data Sheet S1. The one-way ANOVA with type (fast food, Japanese diet, and kitchen utensils) as the within-participants factor showed a significant main effect of type [*F*(2,62) = 16.92, *p* < 0.001, $\eta_{p}^{2}$ = 0.35]. Multiple comparisons revealed that the RTs for fast food [*t*(62) = 5.86, *p* < 0.001] and the Japanese diet [*t*(62) = 3.18, *p* = 0.01] were shorter than those for kitchen utensils. Multiple comparisons also showed that the RTs for fast food were shorter than those for Japanese diet items [*t*(62) = 2.63 *p* = 0.04].

### Accuracy

The accuracy of target detection was high under all conditions (mean ±*SE* %; fast food: 97.4 ± 0.6; Japanese diet: 96.2 ± 0.7; kitchen utensils: 93.9 ± 0.9). The one-way ANOVA with type (fast food, Japanese diet, and kitchen utensils) as the factor showed a main effect of type [*F*(2,62) = 9.68, *p* < 0.001, $\eta_{p}^{2}$ = 0.24]. Multiple comparisons showed that accuracies for fast food [*t*(62) = 3.96, *p* = 0.01] and the Japanese diet items [*t*(62) = 2.68, *p* = 0.04] were higher than those for kitchen utensils, but no difference was detected between the food targets [*t*(62) = 0.80, *p* = 0.25]. These results indicate that the RT results were not attributable to a speed-accuracy trade-off phenomenon. The spherical distribution assumption was confirmed (Mauchly’s test, *p* = 0.17), but the data were not normally distributed. Therefore, we confirmed the effect of food vs. non-food on the accuracy of detection (fast food: *p* = 0.003; Japanese diet: *p* = 0.1) using the Kruskal–Wallis test.

### Rating

The results of the evaluation of the degree of liking and prices of fast food and the Japanese diet items are shown in **Table 1**. A *t*-test showed no difference in the degree of liking for fast food or Japanese diet items [*t*(31) = 1.56, *p* = 0.13]. In contrast, a *t*-test showed that higher prices were associated with the Japanese diet items than with the fast food items [*t*(31) = 3.63, *p* = 0.001].

**Table 1:** Mean ± *SE* of liking ratings and prices of food stimuli.

	Fast food	Japanese diet
Liking^a^	3.95 ± 0.11	4.15 ± 0.09
Price (JPY)^b^	187.80 ± 10.35	223.35 ± 9.90

*^a^Participants rated each food item on a 5-point scale (1 = “dislike very much,” 5 = “like very much”). ^b^Participants evaluated each food item in terms of a reasonable price if they were to pay for it.*

### Relationship between RT and Other Variables

The relationships between detection speed and BMI, subjective hunger levels, subjective ratings of liking, and monetary values of food targets were investigated, but no relationship was found between the RTs under each target condition and BMI (fast food: *r* = 0.01, *p* = 0.96; Japanese diet: *r* = 0.03, *p* = 0.86), or subjective hunger levels (fast food: *r* = 0.23, *p* = 0.21; Japanese diet: *r* = 0.28, *p* = 0.13). We also observed no relationship between RTs and liking (fast food: *r* = -0.03, *p* = 0.88; Japanese diet: *r* = 0.08, *p* = 0.68) or between RTs and price (fast food: *r* = -0.24, *p* = 0.18; Japanese diet: *r* = 0.17, *p* = 0.36).

## Discussion

Our results show that the RTs for detecting both fast food and Japanese diet items were shorter than those for detecting kitchen utensils, indicating that food targets are detected more rapidly than non-food targets. This is consistent with previous results showing that photographs of food were more rapidly detected than were those of non-food items (Nummenmaa et al., 2011; de Oca and Black, 2013). However, these previous results involved comparison of the RTs for detecting food and non-food targets that appeared in the context of target and distractor stimuli (i.e., search asymmetry). In contrast, in the present study, the RTs for detecting food and non-food targets were compared under the same distractor conditions. Relative to previous studies using the search asymmetry paradigm, the present study allowed for direct and more precise assessment of the rapid food detection (Eastwood et al., 2001). Moreover, our result is also in line with another visual search study using food-relevant and irrelevant words that reported that RTs were shorter for targets with food names than for targets with object names within a crowd of other non-food word distractors (Hollitt et al., 2010). However, our results were not consistent with other experiments of previous studies (Nummenmaa et al., 2011; de Oca and Black, 2013) that showed null findings when considering the effect of food vs. non-food on rapid detection. Methodological differences, such as fasting duration and hunger levels, which were manipulated in the present study, may account for the discrepancies across studies. In the current study, the participants were asked to fast for more than 3 h and attend the experimental laboratory once they felt hungry. However, fasting duration or hunger levels were not controlled so as to be identical in previous visual search studies (Nummenmaa et al., 2011; de Oca and Black, 2013). These factors influence the degree of visual attention toward food (Mogg et al., 1998; Tapper et al., 2010; Harrar et al., 2011; Gearhardt et al., 2012) and may facilitate the rapid detection of food vs. non-food. Taken together, our results clearly show that food is more rapidly detected within the environment compared with non-food.

Furthermore, the results showed that the RTs for detecting fast food were shorter than those for detecting Japanese diet, suggesting that high-fat food is detected faster than low-fat food. This result is in line with previous studies showing that people can correctly evaluate the fat content of food based on visual information only (Toepel et al., 2009) and that high-calorie, high-fat food enhance visual attention compared with low-calorie, low-fat food (Toepel et al., 2009; Harrar et al., 2011). Our results are also consistent with the theoretical proposal that fatty food is processed in the brain according to its emotional salience or reward value associated with survival benefit (Rolls, 2000). It is possible that the visual inputs of high fat food are predominantly processed in cortical visual areas, such as the fusiform gyrus (Chen et al., 2016), as well as in subcortical emotion-processing regions, such as the amygdala (Tang et al., 2012), and prefrontal regions, such as the orbitofrontal cortex (Simmons et al., 2005; Tang et al., 2012). However, to date, no empirical study has examined the effect of fat content on the food detection speed. As far as we know, this is the first study to indicate that dietary fat content promotes rapid detection of food.

In contrast to the more rapid detection of fast food than the Japanese diet items, subjective ratings showed that participants liked fast food and the Japanese diet items equally. The prices that they assigned to the Japanese diet items were slightly higher than those of fast food; that is, they assigned a higher economic value to the Japanese diet items than to the fast food stimuli. In terms of the relationship between these subjective ratings and RTs, detection speed was not associated with the evaluations of liking or value. Therefore, our data suggest that high-fat food enhances human visual attention, but this effect might not be modulated strongly by subjective liking or perceived economic value. However, this result was inconsistent with a previous study showing that RTs associated with preferences are faster for more-liked food than less-liked food (Bielser et al., 2016). This discrepancy might be explained by the use of different tasks (i.e., a visual search task vs. task on food choice according to preferences). Further investigation using food items that individuals strongly like and dislike should be conducted to understand the effect of individual differences in food evaluations on the rapidity of detection of food targets.

Our data suggest that both advantages and disadvantages are associated with the system underlying humans’ visual attention to food. The rapid detection of food within an environment plays a crucial role in successful foraging, which decreases the risk of starvation and increases survival chances. Thus, evolution has shaped the human visual system to orient visual attention efficiently to food resources (Spence et al., 2016). Consistent with such evolutionary viewpoints, our results suggest that the rapid detection of food within the environment is based on the need to ingest food for survival and to maintain health. Indeed, long-term collective survival depends on the efficient detection of food and the sensitivity to dietary fat content.

However, nowadays, specifically in Westernized societies, such preferential visual attention to food or to high-fat food is maladaptive, resulting in the overconsumption of dietary fat and increased rates of obesity (Bellisari, 2008). Our investigation of subjective ratings indicated that fast food items promoted intense positive emotions, as with Japanese diet items, although people felt that fast food harmed their health more than the Japanese diet items. The present results concerning the visual search task clearly show that humans have visual systems to efficiently detect food, especially when it contains high levels of fat. This evidence showing that food, especially high-fat food, capture our visual attention preferentially should encourage people to control their fat intake. Individuals who want to restrict their intake of dietary fat should avoid places that feature visual stimuli related to high-fat food, such as food courts and the prepared food areas of supermarkets. Our results indicate that the Japanese diet moderately captures our visual attention but contains low levels of fat, suggesting that the Japanese diet would provide physical and psychological satisfaction in a healthier way than fast food.

Some limitations of this study should be acknowledged. First, we did not observe any significant effects of participant characteristics, such as BMIs, hunger levels, or the degree of liking, on the detection RTs. These null findings might be due to our relatively small sample size or to the narrow ranges of these variables. Further studies on participants with a wider range of BMIs, the hunger-satiated state, and the degree of liking, and a larger sample, would clarify the relationship between these participant characteristics and the rapid detection of food. Second, our preliminary results showed no significant gender difference in visual search performance, but the current study did not have sufficient power to compare between-participant factors. Because some studies have reported a gender difference in cravings for high-fat food (Burton et al., 2007), gender effects on the rapid detection of food and dietary fat content should be clarified by further studies with larger sample sizes. Third, the relationship between RTs for the visual search task and subjective emotional ratings was not investigated in this study. Our additional ratings data show that the fast food and Japanese diet stimuli elicited higher emotional arousal and more positive feelings compared with non-food items, and such rating results are consistent with the results of the visual search task showing facilitation of detection of food vs. non-food targets. It should also be noted that the sample size for the additional ratings data was small (*N* = 16). To understand the psychological mechanisms underlying the rapid detection of food, emotional ratings should be obtained from an identical participant group and with a larger sample size. Fourth, the participants were all Japanese and had extensive experiences of eating the Japanese diet. Although our additional investigation showed no difference in familiarity between fast food and the Japanese diet items, it is possible that cultural differences in eating experiences would modulate the evaluation of food and lead to differences in the rapid detection of food. Additional studies with participants from various cultures would clarify the effects of eating experiences on the detection of food. Fifth, although we controlled for several factors related to the fast food and Japanese diet stimuli (e.g., luminance and nutritional information), other confounding factors may be acting. For example, our ratings experiment, in which the different participant group from that in the visual search experiment participated, showed that the Japanese diet items were more frequently consumed; therefore, the participants might be habituated to the Japanese diet items. Because habituation induces a reduction in behavioral and physiological responses (Epstein et al., 2009), it is possible that habituation to items from the Japanese diet delayed their detection. Further investigations should explore such alternative mechanisms using a different stimulus set. Finally, we did not manipulate stimulus set size, which can be informative regarding search efficiency. When increasing stimulus set size, constant detection RTs imply efficient target detection, whereas longer detection latencies imply inefficient target detection (Wolfe and Horowitz, 2004). Although the present study revealed more rapid detection of food items than non-food items, and of high-fat than low-fat food items, future studies with different stimulus set sizes should be undertaken to further investigate search efficiency of food items.

In summary, two main findings emerged from data gathered from hungry participants with normal weight: both fast food and Japanese diet are detected faster than non-food objects, and fast food with a high fat content accelerates responses compared with Japanese diet. Based on the nourishment and pleasure afforded by food, food preferentially captures visual attention and high fat content further facilitates rapid detection.

## Author Contributions

RS, WS, and TF conceived and designed the study. RS and WS acquired and analyzed data. All authors wrote the manuscript text. RS prepared figures and illustrations of stimuli.

## Conflict of Interest Statement

The authors declare that the research was conducted in the absence of any commercial or financial relationships that could be construed as a potential conflict of interest.

## Supplementary Material

FIGURE S1 Schematic illustration of all stimuli. Actual stimuli were full-color photographs.
