# Supplementary figures and images for: Fat Content Modulates Rapid Detection of Food: A Visual Search Study Using Fast Food and Japanese Diet
Source: Front Psychol. 2017 Jun 22;8:1033. doi: 10.3389/fpsyg.2017.01033 (PMC5479904; doi:10.3389/fpsyg.2017.01033)

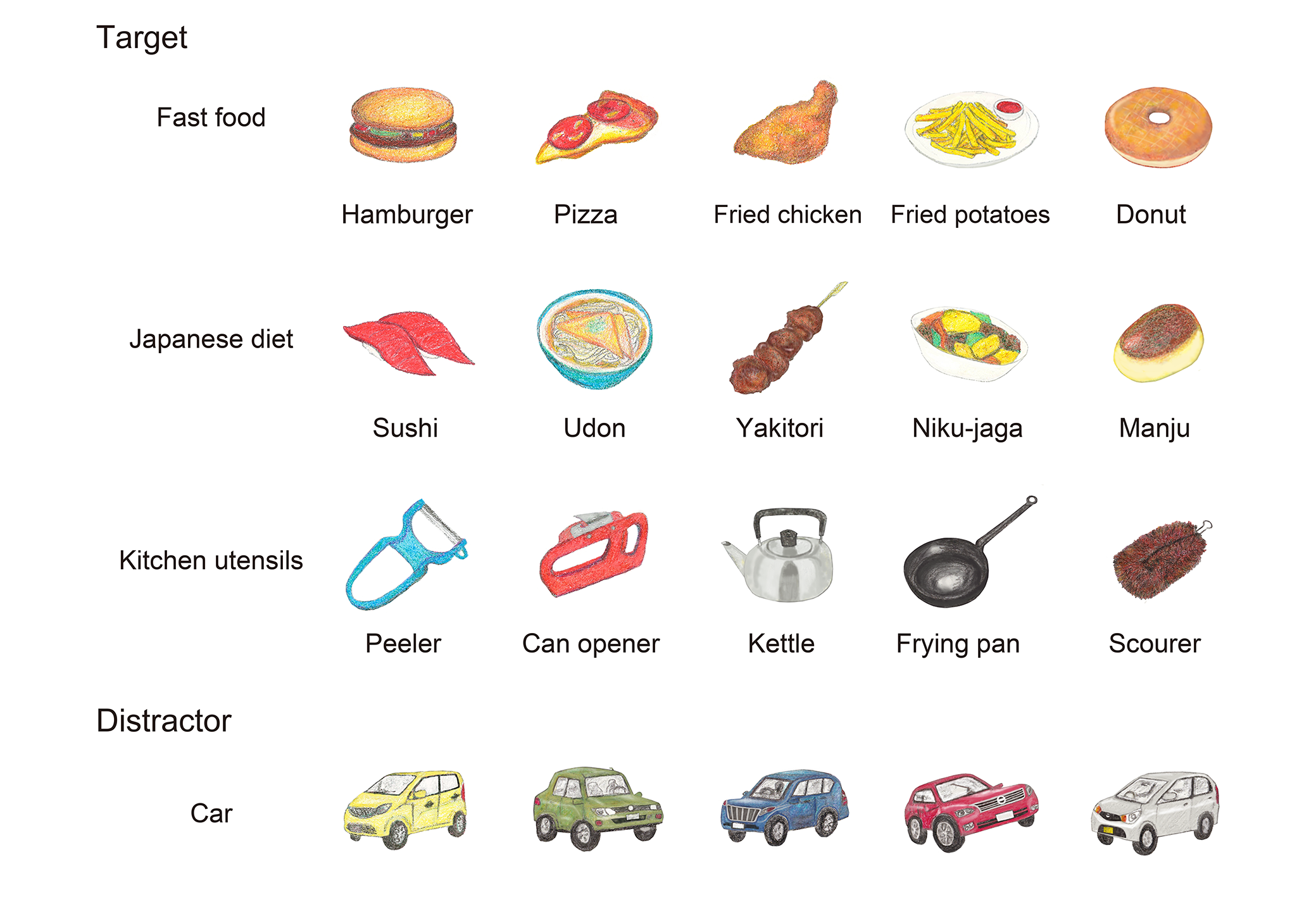

Supplement: FIGURE S1 — Schematic illustration of all stimuli. Actual stimuli were full-color photographs. [file Figure_S1.TIF]
