# Supplementary material for: Fat Content Modulates Rapid Detection of Food: A Visual Search Study Using Fast Food and Japanese Diet
Source: Front Psychol. 2017 Jun 22;8:1033. doi: 10.3389/fpsyg.2017.01033 (PMC5479904; doi:10.3389/fpsyg.2017.01033)
Supplement: Supplementary file 2 [file Table_1.DOC]

**Supplementary Table 1.** Nutritional information for food stimuli.

| Type | Item | Calorie (kcal) | Protein (g) | Fat (g) | Carbohydrate (g) | Salt (g) |
| --- | --- | --- | --- | --- | --- | --- |
| Fast food | Hamburger | 310 | 16.2 | 13.5 | 30.8 | 2.4 |
|  | Pizza | 206 | 9.8 | 9.3 | 20.2 | 1.4 |
|  | Fried chicken | 282 | 14.4 | 16.7 | 18.4 | 1.9 |
|  | Fried potatoes | 237 | 2.9 | 11.0 | 32.0 | 2.0 |
|  | Donut | 234 | 3.7 | 13.6 | 24.0 | 0.6 |
|  | Mean (*SE*) | 253.8 (18.6) | 9.4 (2.7) | 12.8 (1.3) | 25.1 (2.7) | 1.7 (0.3) |
| Japanese diet | Sushi | 88 | 9.0 | 0.6 | 12.0 | 0.6 |
|  | Udon | 344 | 10.5 | 7.4 | 55.4 | 4.7 |
|  | Yakitori | 106 | 10.0 | 5.6 | 3.9 | 0.8 |
|  | Niku-jaga | 341 | 10.3 | 13.1 | 45.0 | 3.8 |
|  | Manju | 124 | 2.4 | 0.5 | 27.3 | 0.01 |
|  | Mean (*SE*) | 200.6 (58.2) | 8.4 (1.5) | 5.4 (2.4) | 28.7 (9.7) | 2.0 (0.1) |
