# Supplementary material for: Fat Content Modulates Rapid Detection of Food: A Visual Search Study Using Fast Food and Japanese Diet
Source: Front Psychol. 2017 Jun 22;8:1033. doi: 10.3389/fpsyg.2017.01033 (PMC5479904; doi:10.3389/fpsyg.2017.01033)
Supplement: Supplementary file 3 [file Table_2.DOC]

**Supplementary Table 2. Mean ± *SE* for emotional arousal, valence, and familiarity scores for target (fast food, Japanese diet, and kitchen utensils) and distractor stimuli (car), and those for eating frequency and healthiness for food stimuli of hungry participants (*N* = 16).**

|  | Fast food | Japanese diet | Kitchen utensils | Car |
| --- | --- | --- | --- | --- |
| Arousal | 6.48 ± 0.29 | 6.29 ± 0.18 | 4.48 ± 0.24 | 4.24 ± 0.29 |
| Valence | 6.46 ± 0.27 | 6.44 ± 0.20 | 4.89 ± 0.16 | 5.15 ± 0.11 |
| Familiarity | 6.56 ± 0.29 | 6.46 ± 0.26 | 5.93 ± 0.26 | 4.79 ± 0.37 |
| Eating frequency | 3.58 ± 0.23 | 4.23 ± 0.12 | - | - |
| Healthiness | 3.74 ± 0.22 | 5.91 ± 0.18 | - | - |
